# Computer copilots for endoscopic diagnosis

**DOI:** 10.1038/s41746-022-00678-7

**Published:** 2022-09-01

**Authors:** James A. Diao, Joseph C. Kvedar

**Affiliations:** Harvard Medical School, Boston, MA USA

**Keywords:** Diagnostic markers, Colorectal cancer, Image processing

## Abstract

Artificial intelligence (AI) tools for endoscopy are now entering clinical practice after demonstrating substantial improvements to polyp detection on colonoscopy. As this technology continues to mature, efforts to develop and validate a new frontier of possibilities—including diagnostic classification, risk stratification, and clinical outcomes assessment—are now underway. In *npj Digital Medicine*, scientists from Cosmo AI/Linkverse and collaborators report an extension to the first FDA-cleared AI tool for colonoscopy that goes beyond polyp detection to enable video-based diagnostic characterization.

Colorectal cancer is among the leading causes of death from cancer in the US^1^ and worldwide^2^, second only to lung cancer. Recommended screening includes colonoscopy, which uses a long, flexible instrument to visualize the colon and rectum. Precancerous growths found during this procedure are sampled or removed. Early detection is critical—patients screened by gastrointestinal (GI) specialists with higher detection rates have lower rates of cancer, advanced-stage disease, and cancer-related death^3^.

In April 2021, the US Food and Drug Administration (FDA) authorized marketing of GI Genius, the first computer-aided polyp detection tool^4^. Developed by Cosmo AI/Linkverse and distributed by Medtronic, GI Genius helps doctors find polyps in real-time by superimposing bright outlines over suspicious lesions on endoscopic video. The agency’s decision was based on a multisite randomized trial demonstrating 30% increased adenoma detection rate relative to unassisted colonoscopy, with no increase in adverse events or resection of benign growths^5^.

Investigating for unnecessary resections was particularly important because they are associated with rare but significant complications—these include bleeding, infection, and life-threatening perforation of the bowel wall. Endoscopists minimize polypectomies by using visual features like size, shape, and vascularity to differentiate benign polyps from their premalignant counterparts. A computer-aided diagnostic tool for this task, termed “optical characterization”, could reduce unnecessary polypectomies while maximizing removal of dangerous growths.

To validate this feature for the newest iteration of GI Genius, Biffi et al. conducted a prospective single-center study comprising 130 patients, each with at least one polyp discovered on AI-assisted colonoscopy^6^. From the resulting video data, 513 videoclips (one per polyp) were extracted and histologically classified as “adenoma,” “non-adenoma,” or “undetermined.” The clips were then reviewed by 10 expert and 11 non-expert endoscopists, who assigned a diagnosis to each videoclip. Experts had performed 500 colonoscopies with at least 6 years of experience, while non-experts had performed 100 colonoscopies with at least 1 year of experience. All endoscopists could view the videos under two settings: unfiltered white light (standard colonoscopy) or filtered blue light (chromoendoscopy, to enhance blood vessels). Their predictions were then compared to those made by GI Genius using either light setting.

GI Genius using white light achieved 85% accuracy, 81% sensitivity, and 87% specificity, comparable to that of experts (82, 77, 87%) and superior to that of non-experts (75, 72, 81%). Although these performance metrics are not nearly sufficient to dispense with histopathologic confirmation, they suggest that computer-aided diagnosis can improve endoscopic performance, especially in settings with fewer highly trained specialists. Notably, most polyps incorrectly classified by GI Genius were also misclassified or disagreed upon by experts. This suggests that computer predictions may be subject to uncertainty and error patterns similar to that of human experts. GI Genius omitted significantly more predictions when using blue light than when using white light (28.1% vs. 6.2%), likely due to decreased information density, but its performance was otherwise unaffected.

The study has limitations. Endoscopists often further classify lesions into more granular diagnostic classes (e.g., hyperplastic, sessile serrated, carcinoma, etc.) than the binary grouping of adenoma versus non-adenoma. They also direct their visual field during endoscopy to gather information based on their real-time diagnostic reasoning, a process which may not be well-represented by post-hoc review of video data. The higher rate of omitted predictions from GI Genius relative to experts and non-experts (6.2% vs. 1.4 and 1.9%, respectively) may also inflate performance metrics, but not enough to affect statistical claims of superiority and non-inferiority.

With more than 15 million colonoscopies performed annually in the US alone^7^, the implications for clinical practice and outcomes are substantial. A modeling study estimated that adding AI support to guideline-based screening for 60% of eligible US adults would cost $250 million annually, but could prevent over 7000 colorectal cancer cases and 2000 deaths every year^8^. After accounting for the cost of missed cancers, net cost savings could surpass $300 million per year^8^. The technology is still early on the adoption curve, but efforts to increase uptake and accessibility are ongoing. These include programs like the Medtronic Health Equity Assistance Program, which recently delivered GI Genius modules to 62 facilities performing colonoscopies in underserved communities^9^.

Cosmo AI/Linkverse is hardly the only company operating in this multibillion dollar market. Other AI systems under development include ENDO-AID by Tokyo-based Olympus^10^, EndoScreener by Shanghai-based Wision AI^11^, Skout by Cambridge-based Iterative Scopes^12^, and Ultivision by Irvine-based DocBot^13^. Many have or may soon be cleared for marketing in the US under the substantial equivalence standard of 510(k), but no company has registered trials to examine patient-centered outcomes like interval cancer.

While it remains possible that the additional computer-detected lesions are predominantly those of little clinical consequence, the preponderance of evidence now signals a powerful and cost-effective tool for standardizing, improving, and extending human capabilities^14^. Quantifying disease severity and progression for GI conditions, such as inflammation in ulcerative colitis^15^, may provide valuable insights for treatment, assessment, and trial design. Beyond colonoscopy, AI could also support endoscopic visualization of the esophagus, stomach, small intestine, and bronchi. In fact, for any unexpected oddity seen through a scope, clinicians may soon be equipped with a new generation of AI tools to help them get to the bottom of it.

## Data Availability

No computer code or datasets were produced or analyzed for this article.
